# The Role of Intraoperative MRI in Awake Neurosurgical Procedures: A Systematic Review

**DOI:** 10.3389/fonc.2018.00434

**Published:** 2018-10-10

**Authors:** Tumul Chowdhury, Frederick A. Zeiler, Gyaninder P. Singh, Abseret Hailu, Hal Loewen, Bernhard Schaller, Ronald B. Cappellani, Michael West

**Affiliations:** ^1^Department of Anesthesiology, Perioperative and Pain Medicine, University of Manitoba, Winnipeg, MB, Canada; ^2^Section of Neurosurgery, Department of Surgery, University of Manitoba, Winnipeg, MB, Canada; ^3^Clincian Investigator Program, University of Manitoba, Winnipeg, MB, Canada; ^4^Division of Anaesthesia, Department of Medicine, Addenbrooke's Hospital, University of Cambridge, Cambridge, United Kingdom; ^5^Department of Neuroanaesthesiology & Critical Care, Neurosciences Centre, All India Institute of Medical Sciences, New Delhi, India; ^6^Max Rady College of Medicine, University of Manitoba, Winnipeg, MB, Canada; ^7^College of Rehabilitation Sciences Librarian, Neil John Maclean Health Science Library, University of Manitoba, Winnipeg, MB, Canada; ^8^Department of Primary Care, University of Zurich, Zurich, Switzerland

**Keywords:** intraoperative magnetic resonance imaging (iMRI), awake craniotomies, outcome, complications, brain tumors

## Abstract

**Background:** Awake craniotomy for brain tumors remains an important tool in the arsenal of the treating neurosurgeon working in eloquent areas of the brain. Furthermore, with the implementation of intraoperative magnetic resonance imaging (I-MRI), one can afford the luxury of imaging to assess surgical resection of the underlying gross imaging defined neuropathology and the surrounding eloquent areas. Ideally, the combination of I-MRI and awake craniotomy could provide the maximal lesion resection with the least morbidity and mortality. However, more resection with the aid of real time imaging and awake craniotomy techniques might give opposite outcome results. The goal of this systematic review.is to identify the available literature on combined I-MRI and awake craniotomy techniques, to better understand the potential morbidity and mortality associated.

**Methods:** MEDLINE, EMBASE, and CENTRAL were searched from inception up to December 2016. A total of 10 articles met inclusion in to the review, with a total of 324 adult patients.

**Results:** All studies showed transient neurological deficits between 2.9 to 76.4%. In regards to persistent morbidity, the mean was ~10% (ranges from zero to 35.3%) with a follow up period between 5 days and 6 months.

**Conclusion:** The preliminary results of this review also suggest this combined technique may impose acceptable post-operative complication profiles and morbidity. However, this is based on low quality evidence, and is therefore questionable. Further, well-designed future trials with the long-term follow-up are needed to provide various aspects of feasibility and outcome data for this approach.

## Introduction

The role of maximal surgical resection in the case of brain tumors especially, intrinsic gliomas has been widely debated in neurosurgery, neuro-oncology, and radiation oncology with the underlying principle of this technique focused of extensive surgical cytoreduction prior to aggressive chemotherapeutic and radiation therapies. Current literature suggests the increasingly strong link between maximal safe surgical resection of high-grade glial neoplasms and both progression free and overall survival (1–8). The role of aggressive surgical approaches for low grade gliomas remains unclear especially in the case of asymptomatic incidental presentations, though some circles argue for similar aggressive resection in younger patient cohorts prior to neuropathologic transformation to high grade lesions (7, 9, 10). In addition, across the spectrum of intrinsic glial tumors, as we begin to better understand the molecular signatures associated with these lesions, it is becoming clearer that certain subtypes of gliomas may benefit from aggressive resection (11–15). However, one must acknowledge the decision to pursue aggressive operative intervention is one that is made weighing the risk and benefit profile, allowing the individual patient to decide what are the “acceptable” risks and potential morbidities.

In order to improve the safety profile associated with extensive surgical resections, awake craniotomy techniques have been implemented, particularly in those lesions located in or near eloquent structures (16). Awake craniotomy is a commonly performed neurosurgical procedure for the resection of brain lesions near to an eloquent area (17–21). This technique increases the safety profile and potentially improves the overall neurological outcome of the patient (17, 18, 20). Importantly, it has become a standard of care in many centers in the world. Such techniques require specialized neurosurgical, neuroanesthesia, and intra-operative neurophysiologic monitoring and nursing personal. The premise relies on the fact that the patient is under “light” sedation throughout the procedure, comfortable enough to tolerate a craniotomy, and tumor resection, but able to awaken and participate in intra-operative clinical examination during resection and direct electrical stimulation of neural structures. This allows the treating team to identify eloquent territories, avoiding aggressive resection in these areas and reducing the risk of permanent post-operative morbidity (22).

In line with the premise of awake craniotomy, intraoperative magnetic resonance imaging (I-MRI) provides real-time imaging and can potentially increase the degree of resection of brain tumors, either identifying missed areas or residual disease not grossly apparent to the neurosurgeon by direct inspection of the surgical field (1, 23). I-MRI has been used in many neurosurgical procedures including primary brain tumor resection, pituitary tumors surgeries and deep brain stimulation for various movement disorders (1, 23–25). In glioma surgery, I-MRI has been employed in patients under general anesthesia in order to optimize surgical results (26–28). To date, literature supports improved resection of gross imaging based T1 and T2 weighted MRI abnormalities. However, under such anesthetic conditions, some argue that with use of I-MRI may increase post-operative transient and permanent morbidity, particularly language and motor deficits (26, 29, 30).

Combining awake craniotomy techniques and I-MRI may provide optimal safe conditions for aggressive surgical resection of intrinsic glial neoplasms. It is plausible that the combination of I-MRI and awake craniotomy can provide maximum tumor resection with less post-operative morbidity and mortality. Therefore, the addition of these two techniques should produce favorable neurological outcomes (31). However, I-MRI assisted maximum resection can also lead to more language deterioration and new neurological deficits (26, 29, 30). In addition, there can be many complications including surgical, anesthetic or radiological during I-MRI use (32–36). Theoretically, combing two techniques may sometimes act as a double-edge sword, and it remains currently unknown the risk profile associated with using both techniques, and it may improve extent of resection at the cost of functional outcomes. Therefore, the goal of this systematic review is to identify the available literature on combined I-MRI and awake craniotomy techniques, to better understand the potential morbidity and mortality associated.

## Methods

### Protocol

This systematic review was registered with PROSPERO, International prospective register of systematic reviews (CRD42016052733). This review involves various steps including preliminary searches, piloting of the study selection process, formal screening of search results against eligibility criteria, data extraction, risk of bias (quality) assessment and data analysis. Though, statistical analysis was not carried out due to heterogeneity.

The protocol is developed on the basis of PICOS [Patient Population or Problem, Intervention (treatment/test), Comparison (group or treatment), Outcomes, and Setting question]. Whether or not, the inclusion of I-MRI with awake craniotomy imposes additional benefit or harm is the basis of this research. This review is reported in keeping with the systematic review guidelines in the preferred reporting in systematic reviews and meta-analysis (PRISMA) statement.

### Search criteria

The search strategy was developed by the primary investigator (TC) in consultation with a professional librarian at Neil John Maclean Health Science Library, Winnipeg, Canada (HL). A search was conducted in the databases: MEDLINE, from 1946 to December 1, 2016 EMBASE, from 1996 to December 2, 2016, and the Cochrane Central Register of Controlled Trials (CENTRAL), issue 11 of 12 (December 1, 2016). The search strategy included appropriate subject headings and keywords for the concepts terms of awake neurosurgical procedure, awake craniotomy, and intraoperative magnetic resonance imaging. There were no language restrictions on the search. The detailed search terms are given in Appendix A in Supplementary Material. The study population of interest included adult patients undergoing awake neurosurgical procedures under I-MRI for brain tumors. Pediatric patients (aged < 18 years), and pregnant patients undergoing the above mentioned procedures were excluded. Retrospective as well as prospective observational studies, randomized clinical trials, and case series involving more than four cases were included for this systematic review.

### Data collection and quality assessment

On the basis of above defined terms, Initial titles and abstracts were provided by HL (librarian). All data (titles, abstract, exclusion criteria) were recorded in Microsoft Excel 15.0 version (password protected). Three separate sheets were created. The first sheet was for the titles and abstracts, second for the screened titles and abstracts (on the basis of inclusion/exclusion criteria) and third one for the final articles (on the basis of full texts). This part of data collection was done by two independent investigators (AH and GP) and any discrepancy was sorted out by the third (TC). In case, if primary or secondary outcomes defined for the project were not mentioned in the articles, corresponding authors were contacted to provide the data or clarification by the principal investigator (TC). The quality assessment was done by two reviewers (AH and GP). We used the Cochrane Collaboration' tool to assess the risk of selection, performance, detection, attrition, and reporting biases. For reducing selection bias, the fourth reviewer (FZ) reviewed all the data provided on sheet 2 and sheet 3 as well as cross-references. All studies were also categorized as direct, if mentioned awake craniotomies as the primary study subjects, and indirect, if mentioned awake craniotomies as one of the parts of total study subjects.

### Outcome(s)

#### Primary outcome(s)

The primary objective of this study is to note the effect of I-MRI on overall morbidity in patients undergoing awake neurosurgical procedures. Morbidity is defined as any new neurological deficit or worsening of pre-existing neurological deficits. This is further divided into two: transient (short term) and persistent (long term). Transient deficits were defined as any morbidity that improved during the study period whereas persistent is defined as any morbidity that persisted through out the study period.

#### Secondary outcome(s)

We noted the effect of I-MRI on various other parameters including extent of resection of brain tumor, intraoperative surgical complications, intraoperative anesthetic complications, intraoperative radiological complications, total duration of procedure and overall mortality.

### Data synthesis

A descriptive data summary is presented as events numbers/proportions/percentages. To explain the data further, various tabulated aspects are presented in Tables 1–5. No formal statistical analysis was done. Meta-analysis was not carried out, as we did not have sufficient homogenous data, and there were lack of randomized controlled trials.

**Table 1:** Study characteristics and level of evidence.

**References**	**Study Type**	**Level**	**SubjectsI-(n)**	**MRI**	**Volumetric Analysis**	**Objective**	**Follow up criteria**
Nabavi et al. (37)	R, D	IV	34^*^	1.5T	N	Feasibility, Adverse events	NA
Weingarten et al. (38)	P, D	IV	10	1.5T	N	Feasibility of integration of neuronavigation and electrostimulation with I-MRI	NA
Goebel et al. (39)	P, D	IV	25	1.5T	N	Patients' perception	5 days
Leuthardt et al. (40)	R, D	1V	12	1.5T	N	EOR, Functional outcome	1 month
Lu et al. (41)	P,D	IV	30	3T	Y	EOR, Functional outcome	6 months
Tuominen et al. (42)	R (CC), D	III	20	0.23T	N	Functional outcome	2 months
Maldaun et al. (43)	R, D	IV	41^**^	1.5T	Y	Feasibility, EOR, Functional outcome	1 month
Zhuang et al. (44)	R, In	IV	20	3T	Y	Feasibility, EOR, Functional outcome	6 months
Coburger et al. (45)	MR, In	IV	9, 17	0.2T, 1.5T	N	Functional outcome, PFS	3 months
Ghinda et al. (46)	R, D	IV	106	3T	Y	Functional outcome, PFS, EOR	1^a^ month

T, tesla, I-MRI, intraoperative magnetic resonance imaging; n, number; R, retrospective; P, prospective, CC, case control, MR, multicenter retrospective, D, direct, In, indirect, EOR, extent of resection, NA, not available, PFS, progression free survival, N, No, Y, Yes,

* *Number of patients were 34 (number of procedures-38)*,

** *Number of patients 41 (number of procedures-42)*,

a *Average follow up period was 24. 8 months but criteria to divide transient to persistent was one month*.

## Results

### Study selection and characteristics

**Figure 1:**
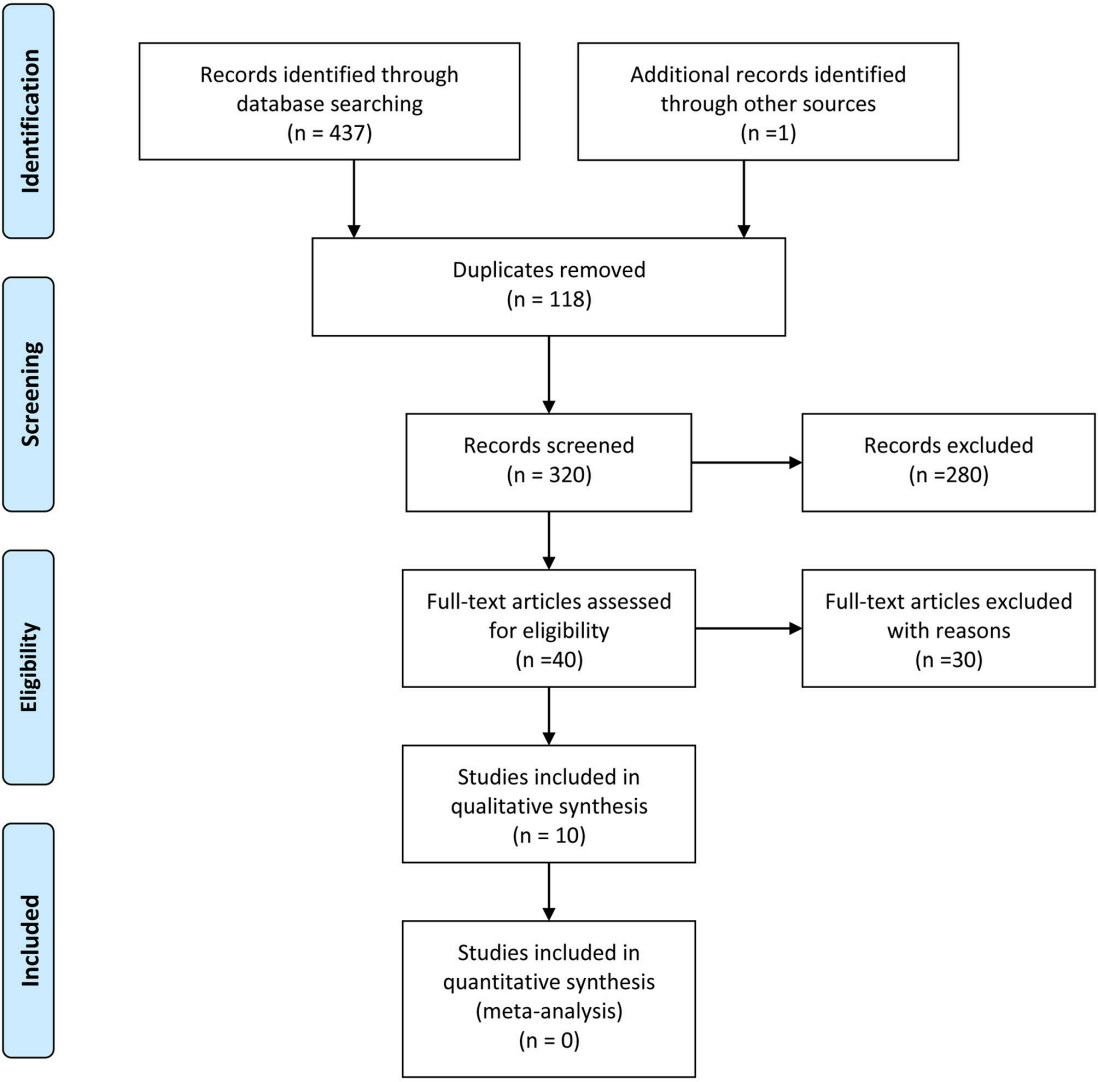
PRISMA flow chart.

Our search strategy retrieved 438 titles and abstracts, and the subsequent filtering process is presented as a PRISMA flow chart [Figure 1]. After the deletion of duplicate results, 320 titles and abstracts were selected. Out of these, 280 articles were excluded on the basis of the pre-defined inclusion and exclusion criteria, 40 were screened further. After going through full texts for all 40 articles, only 10 articles met the criteria, and selected for final inclusion (37–46). All studies were conducted in a single center except one that involved 6 German centers (45). Seven were retrospective and 3 were prospective studies (Table 1). Only one study included a control group [craniotomy under general anesthesia]. Three articles discussed 3 Tesla (T) I-MRI, five articles 1.5 T, two articles 0.2 T and one article mentioned both 0.23 T and 1.5 T. All articles showed level IV evidence except one that had a level III evidence. All studies were published between 2008 and 2016. These included 324 patients. Most of the studies primarily aimed at exploring the feasibility, functional outcome, and extent of resection. Few highlighted the progress free survival, adverse events and patient perception. Only four articles conducted the volumetric assessment for tumors.

### Outcome results

We included all those studies that mentioned morbidity data (Table 2). All studies had mentioned transient and persistent morbidity. All studies showed transient neurological deficits (speech disturbances, and/or motor weakness, and/or sensory deficits) between 2.9 and 76.4% with a mean of 35.6%. In regards to persistent morbidity, the mean was ~10% (ranges from zero to 35.3%) with a follow up period between 5 days and 6 months. Two studies failed to disclose the exact follow-up duration.

**Table 2:** Primary outcome (s) in patients undergoing awake craniotomies under I-MRI.

**References**	**Age Median (range)**	**Demographics n (M, F)**	**I-MRI strength (Tesla)**	**Anesthetics**	**Morbidity (Primary outcome) (neurological deficits %)**
Nabavi et al. (37)	42 (23–69)	34 (20 M, 14 F)	1.5 T	P+R	Transient-2.9a, Persistent-no
Weingarten et al. (38)	41 (25–57)	10 (6 M, 4 F)	1.5 T	Sedation (NA)	Transient-25b, Persistent-no
Goebel et al. (39)	46.2 (23–71)	25 (14 M, 11 F)	1.5 T	P+R	Transient-28c, Persistent-32d
Leuthardt et al. (40)	41 (32–60)	12 (9 M, 3 F)	1.5 T	P+D+A (AWA)	Transient-41.6e, Persistent−25f
Lu et al. (41)	45.5 (19–75)	30 (21 M, 9 F)	3 T	M+D+R+P	Transient-40b, Persistent-3.3b
Tuominen et al. (42)	44 (16–67)	20 (9 M, 11 F)	0.23 T	P+F	Transient-10b, Persistent-10g
Maldaun et al. (43)	41 (22–70)	41 (25 M, 16 F)	1.5 T	P+R+D (AWA)	Transient-26.2^*^, Persistent-2.4^*^
Zhuang et al. (44)	42 (26–62)	20 (13 M, 7 F)	3 T	M+D+R+P	Transient-55.5b, Persistent-5.6b
Coburger et al. (45)	NA	9 (NA)	0.2 T	NA	Transient-33.3^*^, Persistent−11.1^*^
		17 (NA)	1.5 T	NA	Transient-76.4^*^, Persistent-35.3^*^
Ghinda et al. (46)	41.7 (18–76)	106 (74 M, 32 F)	3T	P+D+R	Transient-46^*^, Persistent-8.7^*^

T, tesla; I-MRI, intraoperative magnetic resonance imaging; n, number; (M; F), (Male; Female); P, propofol; R, remifentanil; NA, not available; D, dexmedetomidine; A, alfentanil; AWA, asleep wake asleep; M, midazolam; F, fentanyl; ^a^right arm weakness; ^b^speech problems; ^c^All patients had preoperative deficits; ^d^one of the deficits (motor; speech or sensory); ^e^4 patients had word-finding difficulties; one had left sided inattention; ^f^one left-sided weakness and two had word-finding difficulties; ^g^one patient developed both aphasia and hemiparesis and other had hemiparesis;

* *Either speech problems or motor deficits or both*.

For the secondary outcomes, 9 studies reported percentage of patients with gross total resection (15–70%) on final scans, however, only four included the GTR information (5–36.7%) after the first scan (Table 3). Among intraoperative complications, eight studies noted surgical complications whereas anesthetic as well as radiological problems were mentioned in a single study (Table 3). Majority of the surgical complications included seizures during cortical stimulations. Only, one study reported the mortality in one patient (44). Imaging, operative, and tumor information are also presented; however, these data are quite variable (depend upon institutional and I-MRI characteristics) and preclude any relevant interpretation (Tables 4, 5). Along with I-MRI, all studies have utilized multi-modal monitoring techniques to further localize tumors (Table 5).

**Table 3:** Secondary outcome(s) including resection of tumor, intraoperative complications and mortality in patients undergoing awake craniotomies under I-MRI.

**References**	**I-MRI**	**Patients (%) with GTR**		**Intraoperative complications (n)**				**Mortality**
	**Strength**	**First Scan**	**Final Scan**	**Anesthetic**	**Surgical**	**Radiological**	**Excluded**	
Nabavi et al. (37)	1.5T	NA	NA	None	3^*^	None	1 (postictal paresis)	NA
Weingarten et al. (38)	1.5T	10	70	None	none	None	None	NA
Goebel et al. (39)	1.5T	NA	56	1	5+	1	3 (no I-MRI)	0
Leuthardt et al. (40)	1.5T	8.3	42	None	None	None	None	NA
Lu et al. (41)	3T	36.7	60	None	4^*^	None	None	0
Tuominen et al. (42)	0.23T	NA	50	None	2!	None	None	0
Maldaun et al. (43)	1.5T	24	40.5	none	3^*^	None	None	NA
Zhuang et al. (44)	3T	5	15	NA	4^*^	NA	2 (PH)	1
Coburger et al. (45)	0.2T	NA	NA	NA	1#	NA	None	NA
	1.5T	NA	NA	NA	5∧	NA	None	NA
Ghinda et al. (46)	3T	NA	60.4	None	4^*^	None	2 (no follow up)	NA

T, tesla; I-MRI, intraoperative magnetic resonance imaging; n, number; GTR, gross total resection; NA, not available; S, seizure; PH, postoperative hematoma;

* *three patients had seizures during cortical stimulation (out of these; one developed post-ictal right arm weakness); ^+^two patients had seizures during cortical stimulation; one had intracranial hemorrhage; one had infarct and one had brain swelling; ^!^one patient had seizure during stimulation; other had seizure but not during stimulation; ^#^one patient had intracranial hemorrhage; ∧one patient had ischemia; three patients developed neurological deficits; and one had intracerebral hemorrhage*.

**Table 4:** Imaging and operative characteristics in patients undergoing awake craniotomies under I-MRI.

**References**	**I-MRI**	**Pre-op scans (n)**	**Imaging**	**Scans**	**Scan time (min)**	**Patients [n (%)] with further resection**	**Operation time (h)**
Nabavi et al. (37)	1.5 T	Y (−1, 0)	T1, T2 (i), C	NA	20–60	NA	NA
Weingarten et al. (38)	1.5 T	Y (−1, 0)	T1, T2, C	1–3	30–40	7	6.8 (3.8–8.7)
Goebel et al. (39)	1.5 T	Y (−1, 0)	T1, T2 (i), C	0–2	NA	20	4.8 (3.5–6.75)
Leuthardt et al. (40)	1.5 T	NA	T1, T2, C	1	48–75	6	4.76 (2.7–6.0)
Lu et al. (41)	3 T	Y (−1)	Various, C	NA	NA	11	NA
Tuominen et al. (42)	0.23 T	Y	NA	NA	NA	NA	4.5 (3.2–7.5)
Maldaun et al. (43)	1.5 T	Y	Various, C	NA	5.3–58	7	7.3 (4–13.9)
Zhuang et al. (44)	1.5 T	Y	Various, C	1–3	40	7	NA
Coburger et al. (45)	0.2 T, 1.5 T	NA	NA	NA	NA	NA	NA
Ghinda et al. (46)	3T	Y (−1)	Various, C	1–2	NA	30	NA

*T, tesla, I-MRI, intraoperative magnetic resonance imaging; n, number, NA, not available; min, minutes; h, hours; Y, yes, (−1, 0), 1 day prior and same day; C; contrast, T2 (i), T2 sequence for the initial scan*.

**Table 5:** Tumor characteristics, number of patients with pre-operative deficits or symptoms, number of patients with redo-operations and intraoperative localization techniques during awake craniotomies under I-MRI.

**References**	**Patients (*n*)**	**I-MRI**	**Tumor type**	**Laterality**	**Preop-deficits**	**Localization Techniques**	**Redo operations plus biopsies (n)**
Nabavi et al. (37)	34^*^	1.5 T	Unknown Glial	32-L, 6-R	NA	Cortical stimulation	4
Weingarten et al. (38)	10	1.5 T	Unknown primary	6-L, 4-R		Cortical stimulation, MRI Neuronavigation	0
Goebel et al. (39)	25	1.5 T	Glial (WHO I-IV)	22-L, 3-R	19	Electrical stimulation, MRI Neuronavigation	10
Leuthardt et al. (40)	12	1.5 T	Glial (WHO II-IV)	9-L, 3-R	1	Cortical stimulation, MRI Neuronavigation	4
Lu et al. (41)	30	3 T	Glial (WHO II-IV)	30-L	8	Electrical stimulation, MRI Neuronavigation	5
Tuominen et al. (42)	20	0.23 T	Glial (WHO I-IV)	13-L. 7-R	12	Electrical stimulation, MRI Neuronavigation, F-MRI, USG	8
Maldaun et al. (43)	41^**^	1.5 T	Glial (WHO II-IV)	31-L, 11-R	9	Electric stimulation, MRI Neuronavigation, DTI Tractography	6
Zhuang et al. (44)	20	1.5 T	Glial (WHO II-IV)	20-L	3	Electrical stimulation, Functional MRI, MEPs, MRI Neuronavigation, DTI Tractography	2
Coburger et al. (45)	9	0.2 T	Glial (WHO II)	NA	6	Unknown	NA
	17	1.5 T	Glial (WHO II)	NA	12	Electric stimulation, USG	NA
Ghinda et al. (46)	106	3T	Glial (WHO II-IV)	94-L, 12-R	56	cortical stimulation, MEPs, MRI Neuronavigation, DTI Tractography	NA

T, tesla; I-MRI, intraoperative magnetic resonance imaging; n, number; GTR, gross total resection; NA, not available; L, left; R, right; MEPs, motor evoked potentials; USG, ultrasound; DTI, diffuse tensor imaging; WHO, World Health Organization; F-MRI, functional magnetic resonance imaging;

* *number of patients were 34 (number of procedures-38)*,

** *number of patients 41 (number of procedures-42)*.

## Discussion

Our review of the literature on awake craniotomy plus I-MRI in the resection of intrinsic brain tumors has yielded important results, which deserve highlighting.

First, regarding the primary outcome of patient morbidity, the cumulative results are in keeping with literature on those patients undergoing resection of eloquently located glial neoplasms in the absence of awake craniotomy or I-MRI (17, 18, 47–49). Thus, from the 10 studies included within this review, the combined use of awake craniotomy techniques with I-MRI may not increase the post-operative transient and persistent neurological morbidity, with the range identified from 2.9 to 76.4% and 0 to 35.3%, for transient and persistent morbidity respectively. Though, it should be acknowledged that the overall patient numbers for all included studies are low, given the complexities of such techniques and the need for costly equipment. In addition, the studies suffered from a global lack of controls for comparison, in the setting of heterogeneous pathology, location, surgical teams/techniques, and I-MRI types/field strength. Thus, one must be reserved in implying that the combination of awake craniotomy and I-MRI is equivalent in safety to resection in the absence of such techniques, or in the presence of either only awake craniotomy or I-MRI. Therefore, the results of this systematic review provide preliminary evidence only to support safety, with much further investigation required to demonstrate equivalence or superiority. Furthermore, it must be emphasized that the use of these techniques, awake craniotomy and I-MRI, are typically reserved for those patients with eloquently located intrinsic tumors, as was the case for all studies included in this review. As such, the expected post-operative morbidity for resections carried out in such territories is high, and not necessarily a reflection of the combined technique, but the risk of operating in such cortical areas. Furthermore, as we've demonstrated, despite relatively high transient post-operative morbidity, these deficits typically resolve quickly during follow-up.

Second, with the application of this combined technique, the extent of GTR appears to be in keeping with standard I-MRI studies, where patients were under general anesthesia (17, 20, 21) This result provides preliminary evidence to support the notion that awake craniotomy techniques during I-MRI cases do not limit the ability to obtain acceptable operative resections for intrinsic tumors. With appropriate anesthetic techniques, one can perform similar resections to patients under general anesthetics. Though based on the small patient numbers in the included studies, these comments should be considered preliminary, with further investigation required.

Third, the surgical complication profile for these studies is in keeping with that described in other glioma surgical series and I-MRI series (17, 36, 50). This patient population classically carries a high pre-operative rate of epilepsy, and intra-operative rate of seizures. Our review demonstrated almost all intra-operative surgical complications were seizures, a well-described complication of cranial surgery, especially in cortically located intrinsic tumors. Thus, the combined technique of awake craniotomy and I-MRI does not appear to increase the intra-operative surgical complication profile. Though one must acknowledge, the use of I-MRI requires extensive surgical team training prior to implementation. Furthermore, the use of awake craniotomy techniques is also a specialized skill set, requiring collaborative efforts between the neurosurgical and neuroanesthesia teams. The appropriate awake craniotomy techniques are acquired through specialized training and require both knowledgeable and attentive teams to carry out successfully for extended duration cases, such as the resection of eloquently located intrinsic tumors while using I-MRI. Thus, the low surgical complication profiles seen in the studies included in this review are likely a reflection of the highly trained teams involved in these operative cases. This is also emphasized by the lack of operative mortality within the described studies.

Fourth, the overall operative durations, when reported, ranged from 2.7 to 13.9 h. This time is including the additional time required for I-MRI scan acquisition. As every tumor is a different entity, it can be difficult to provide hard guidelines on the expected duration for the resection of such lesions. In general, for the resection of eloquently located intrinsic tumors, this operative range is in keeping with other series where the combined awake craniotomy/I-MRI technique is not utilized (36). Thus, based on the small cohorts described in the parent studies included in this review, it appears that the overall operative times are not dramatically increased secondary to the application of this combined approach.

Fifth, one potential concern regarding I-MRI remains various radiologic complications including dye induced adverse reactions and anaphylaxis, image distortions, burn injury, interference with anesthetic monitors, and failure to complete the scan. Our review demonstrated only one complication. This complication was a technical one, precluding scanning, resulting in no direct patient related consequences (38). As such, with the appropriate training and safety precautions, I-MRI in the presence of awake craniotomy techniques, can be safely conducted.

Finally, meticulous anesthetic techniques and medications have provided a safe environment for carrying out these prolonged and complex neurosurgical cases under IMRI. Majority of centers have utilized a combined approach of nerve blocks, local anesthetic infiltration and sedation (37–39, 41, 42, 44–46). Two centers have used general anesthesia (deep sedation) with supra-glottic airway device, laryngeal mask airway to protect the airway during initial and later phases of the procedure, and patients were subsequently awaken during the stimulation and tumor excision phase (40, 43). Only one study had reported an anesthetic complication intraoperatively (39). Notably, very few patients showed agitation, fatigue and non-compliant with the procedure in this study; however, there was no robust study designed exclusively for these parameters (39). Therefore, it is apparent that the present anesthetic techniques with standard monitoring make this challenging procedure safe and comfortable to the patients.

### Limitations

Despite the interesting results generated from this systematic review, there are some important limitations that deserve highlighting.

First, the overall number of studies where awake craniotomy techniques in combination with I-MRI were used is quite small, at 10 studies identified. Furthermore, most studies focused on small patient populations with heterogeneous patient characteristics, tumor locations and histopathology. As a result, the overall conclusions regarding this combined operative technique for the resection of eloquently located intrinsic brain tumors are limited. Subsequently, the results of this review should be considered preliminary; supporting the need for properly designed prospective studies into the use of such techniques in glioma surgery.

Second, patient morbidity post operatively is influenced by various factors. Such factors include tumor location, pre-operative deficits, extent of resection, tumor biology, duration of follow-up and also, surgical experiences. The studies included were all focused on eloquently located lesions, however, the location and extent of such lesions varied significantly. In addition, the extent of pre-operative deficits was also heterogeneous. Extent of resection is influence by numerous factors, which will be discussed below. With that said, post operative morbidity is intimately linked with the extent of resection for eloquently located intrinsic tumors. Furthermore, tumor biology is important to acknowledge. The tumor histopathologic grade carries important implications for post-operative clinical course and the use of adjunctive chemotherapeutic and radiation techniques. Higher grade lesions tend to have a more complicated post operative and follow-up course, impeding the ability to determine if persistent deficits are related to surgical resection, inherent tumor biology or secondary effects of chemotherapeutic and radiation therapies. Finally, the duration of follow-up is important. The overall follow-up duration in the included studies ranged from 5 days to 6 months. Thus, any deficits seen during these periods may be permanent or in the process of ongoing evolution. It is difficult to comment on operative morbidity accurately with such heterogeneous and short follow-up periods.

Third, the GTR rates described within the included studies is subject to numerous factors. These factors include pre-operative expectations for resectability, patient/surgeon threshold for “satisfactory” and “acceptable” outcome, type of I-MRI used, and the use of various other intra-operative surgical adjuncts. Based on tumor location, size and extension, there is usually a pre-operative notion of how resectable an intrinsic lesion will be. These views based on pre-operative imaging likely continue to influence an individual surgeon's willingness to continue aggressive resection, and the pre-determined goal of a given operation (i.e., “GTR” or subtotal resection). Further, based on pre-operative clinical phenotype of the patient and both the surgeon/patient's view on what is an “acceptable” outcome, the extent of surgical resection of intrinsic brain tumors is dictated by such notions. What is deemed “acceptable” for outcome and morbidity varies significantly from patient to patient, and from surgeon to surgeon. As such, the GTR rates in this review are also likely a reflection of this. In addition, the type of I-MRI utilized can influence the ability to obtain GTR. Low field strength I-MRI was demonstrated to be inferior to high field (i.e., 1.5 or 2T) in the ability to obtain GTR in one study (45). Thus, comparing the resection rates for low and high field I-MRI is controversial, given the information provided by such low field units is inferior. Finally, many of the studies describe the application of various other intra-operative surgical adjuncts to aid with resection, including: MRI neuronavigation, DTI tractography, pre-operative fMRI, electrophysiology including cortical mapping, and intra-operative ultrasound. All of these adjuncts aid with localization of tumor and eloquent cortex. Thus, the GTR rates, patient morbidity and operative complication profiles described within this review are likely influenced by all of these factors, making the exact impact of awake craniotomy/I-MRI on these outcomes difficult to discern.

Finally, and arguably the most important, is to re-emphasize that the comments and conclusions of this review should be considered preliminary. Based on the individual limitations highlighted above and the small patient numbers, one should be cautioned into considering the combination of awake craniotomy and I-MRI to be equivalent to standard glioma resection techniques, in the presence or absence of awake craniotomy or I-MRI alone. The significant heterogeneity in patients, pathology, lesion location, surgical teams, resection techniques, equipment, field strength, and follow-up information makes the results presented here preliminary for the combined efforts of awake craniotomy and I-MRI for glioma surgery. This is despite the data suggesting safety and comparable extent of resection and peri-operative complication profiles, in comparison to the existing literature. Much further work is required to investigate this combined technique, employing multi-center studies with control subjects and standardized surgical techniques, I-MRI technology and clinical follow-up principles.

## Conclusion

This systematic review suggests that the awake craniotomy combined with intraoperative MRI is feasible and safe to conduct. The preliminary results of this review also suggest this combined technique may impose acceptable post-operative complication profiles and morbidity. However, this is based on low quality evidence, and is therefore questionable. Further, well-designed future trials with the long-term follow-up are needed to provide various aspects of feasibility and outcome data for this approach.

## Author contributions

TC developed the hypothesis, assisted substantially in data collection, screening, reviewing, analyzing, contacting the authors, compiling, and writing the manuscript. FZ reviewed and screened the data, and helped substantially in writing the manuscript. GS collected and screened the data and helped writing the manuscript. AH assisted in collecting the data. HL provided the initial data, abstracts, full-texts and formulated the search tables and prisma flow chart. BS assisted in writing, reviewing and editing the manuscript. RC assisted in developing the hypothesis, reviewing and editing the manuscript. MW assisted in writing and editing the manuscript.

### Conflict of interest statement

TC served as a guest associate editor for Frontiers in Neurosciences and is presently serving as an associate editor for Intensive Care Medicine and Anesthesiology section in Frontiers in Medicine. FZ has received salary support for dedicated research time, during which this manuscript was completed. Such salary support came from: the Cambridge Commonwealth Trust Scholarship, the Royal College of Surgeons of Canada—Harry S. Morton Travelling Fellowship in Surgery and the University of Manitoba Clinician Investigator Program. FZ's research is also supported through the Thorlakson Chair in Surgical Research Establishment Grant. BS served as a guest associate editor for Frontiers in Neurosciences. The remaining authors declare that the research was conducted in the absence of any commercial or financial relationships that could be construed as a potential conflict of interest.
