# Supplementary material for: The Role of Intraoperative MRI in Awake Neurosurgical Procedures: A Systematic Review
Source: Front Oncol. 2018 Oct 10;8:434. doi: 10.3389/fonc.2018.00434 (PMC6191486; doi:10.3389/fonc.2018.00434)
Supplement: Supplementary file 1 [file Data_Sheet_1.docx]

Appendix A: Searched Terms

| **#** | **Searches** | **Results** | **Annotations** |
| --- | --- | --- | --- |
| 1 | neurosurgical procedures/ or exp craniotomy/ | 37521 |  |
| 2 | Neurosurgery/ | 15956 |  |
| 3 | (carniotomy or neurosurg*).tw,kw. | 43444 |  |
| 4 | or/1-3 | 77294 |  |
| 5 | (awake or conscious).tw,kw. | 65501 |  |
| 6 | 4 and 5 | 958 |  |
| 7 | ((intracranial or intra-cranial or intracerebral or intra-cerebral) adj3 (operat* or surgical or surger*)).kw,tw. | 3805 |  |
| 8 | 5 and 7 | 33 |  |
| 9 | 6 or 8 | 970 |  |
| 10 | exp Magnetic Resonance Imaging/ | 396990 |  |
| 11 | (MRI or magnetic resonance imag*).tw,kw. | 315526 |  |
| 12 | or/10-11 | 505938 |  |
| 13 | exp Intraoperative Period/ | 19816 |  |
| 14 | (intraoperative or intra-operative).tw,kw. | 106059 |  |
| 15 | or/13-14 | 117005 |  |
| 16 | 12 and 15 | 7941 |  |
| 17 | 9 and 16 | 124 |  |
| 18 | exp Animals/ not (exp Animals/ and Humans/) | 4669483 |  |
| 19 | 17 not 18 | 124 |  |
| 20 | Adolescent/ not (exp Adult/ and Adolescent/) | 565754 |  |
| 21 | exp Child/ not (exp Adult/ and exp Child/) | 1140354 |  |
| 22 | exp Infant/ not (exp Adult/ and exp Infant/) | 797428 |  |
| 23 | or/20-22 | 1775012 |  |
| 24 | 19 not 23 | 121 |  |
